# The Dental Congress

**Published:** 1893-02

**Authors:** 


### The Dental Congress.

Attention is called to the following relating to the duties of the Committee on Invitation and the Committee on Membership:

It will be seen that membership is clearly defined both for this and foreign countries. Read carefully.

### DUTIES OF COMMITTEE ON INVITATION.

The duties of the Committee on Invitation shall be to invite such scientific persons residing in the United States and foreign countries who are not members of the profession, but who by their recognized attainments in special departments of science, would add interest to the meeting. They shall also furnish the Committee on Membership with a list of the names and residences of those invited.

### THE COMMITTEE ON INVITATION.

Have the authority to invite such dentists of high standing and reputation in foreign countries as may be agreed upon by a majority of the committee, and a card from the chairman of said committee to the Chairman of the Committee on Registration shall be deemed evidence of the reputability of the holder thereof to entitle him to membership in the congress.

### DUTIES OF COMMITTEE ON MEMBERSHIP.

The duties of Committee on Membership shall be to pass upon all applications for membership which may be referred to them by the Committee on Registration or the Treasurer.

### MEMBERSHIP.

The membership shall consists of legally qualified and reputable dentists (as defined in the Code of Ethics of the American and Southern Dental Association) residing in the United States, and all foreign dentists regularly qualified by the laws of the countries from which they come, and such other scientific persons as may be invited by the Committee on Invitation. Each and every member to be entitled to one copy of the transactions.

All dentists residing in foreign countries who desire to acquire membership in the congress will file their application with the

Honorary President or Vice-Presidents of their respective countries who are empowered to pass upon their eligibility.

When applications are satisfactory to the Honorary President and Vice-Presidents or a majority of them in said country the names so agreed upon shall be transmitted by July 15, 1893, to the Chairman of the Committee on Registration who will proceed to issue a membership card without further reference, whether they reside in the United States or elsewhere.

